# NF-κB mediates the transcription of mouse calsarcin-1 gene, but not calsarcin-2, in C2C12 cells

**DOI:** 10.1186/1471-2199-8-19

**Published:** 2007-03-06

**Authors:** Heng Wang, Shulin Yang, E Yang, Zhengmao Zhu, Yulian Mu, Shutang Feng, Kui Li

**Affiliations:** 1 Department of Gene and Cell Engineering, State Key Laboratory of Animal Nutrition, Institute of Animal Science, Chinese Academy of Agricultural Sciences, Beijing 100094, P. R. China; 2 Key Laboratory of Animal Genetics, Breeding and Reproduction of the Ministry of Education of China, Huazhong Agricultural University, Wuhan 430070, P. R. China

## Abstract

**Background:**

The calsarcins comprise a novel family of muscle-specific calcineurin-interaction proteins that play an important role in modulating both the function and substrate specificity of calcineurin in muscle cells. The expression of calsarcin-1 (CS-1) is restricted to slow-twitch skeletal muscle fibres, whereas that of both calsarcin-2 (CS-2) and calsarcin-3 (CS-3) is enriched in fast-twitch fibres. However, the transcriptional control of this selective expression has not been previously elucidated.

**Results:**

Our real-time RT-PCR analyses suggest that the expression of *CS-1 *and *CS-2 *is increased during the myogenic differentiation of mouse C2C12 cells. Promoter deletion analysis further suggests that an NF-κB binding site within the *CS-1 *promoter is responsible for the up-regulation of *CS-1 *transcription, but no similar mechanism was evident for *CS-2*. These findings are further supported by the results of EMSA analysis, as well as by overexpression and inhibition experiments in which NF-κB function was blocked by treatment with its inhibitor, PDTC. In addition, the overexpression of NFATc4 induces both the *CS-1 *and *CS-2 *promoters, whereas MEF2C only activates *CS-1*.

**Conclusion:**

Our present data suggest that NF-κB is required for the transcription of mouse *CS-1 *but not *CS-2*, and that the regulation of the calsarcins is mediated also by the NFAT and MEF2 transcription factors. These results provide new insights into the molecular mechanisms governing transcription in specific muscle fibre cells. The calsarcins may also serve as a valuable mechanistic tool to better understand the regulation of calcineurin signalling during muscle differentiation.

## Background

Calcineurin, a calcium/calmodulin-dependent serine threonine phosphatase, is an important signalling molecule in skeletal muscle, as it promotes differentiation, the slow-fibre phenotype, and possibly also fibre hypertrophy. Calcineurin binds to the calsarcins, a family of muscle proteins that are specific to the sarcomeric Z-disc, which is a focal point in the regulation of contraction both in skeletal and cardiac muscle. Calsarcin-1, -2 and -3 all interact with calcineurin and the Z-disc proteins α-actinin, γ-filamin, myotilin, telethonin and cipher [1]. Several groups have independently identified the calsarcin family and termed it calsarcin [2], FATZ [3], myozenin [4], and c4orf5 [5], in separate reports. The calsarcins may also have a structural role in Z-disc assembly via their ability to bind different Z-disc proteins, as well as a possible involvement in signalling pathways that are activated via their binding to calcineurin.

CS-1 is the only member of the calsarcin family that is expressed in the adult heart and in slow-twitch skeletal muscle, whereas CS-2 and CS-3 are expressed in fast-twitch muscle [2,6,7]. Several studies have also shown that calcineurin controls the skeletal muscle fibre type by stimulating slow muscle gene promoters and slow fibre differentiation both in cultured cells and in vivo [8,9]. In addition, CS-1 knockout mice show enhanced calcineurin signalling and an excess of slow skeletal muscle fibres, indicating that CS-1 negatively modulates the function of calcineurin [10]. Given that both the functions and substrate specificity of calcineurin are modulated by the calsarcins, and that calcineurin operates in the signalling pathways that control the muscle fibre type, a mechanism of muscle fibre determination and differentiation that is regulated by the interactions between calcineurin and the calsarcins can possibly be hypothesized. However, whether the fibre type-specific expression of calsarcins is also controlled by calcineurin remains to be determined.

In our current study, we have investigated the molecular mechanisms underlying the transcriptional regulation of the mouse *CS-1 *and *CS-2 *genes, with a particular focus on the promoter sequences of these genes as they are responsive to calcineurin signalling.

## Results

### Determination of transcription start sites (TSS) for mouse *CS-1 *and *CS-2*

**Figure 1:**
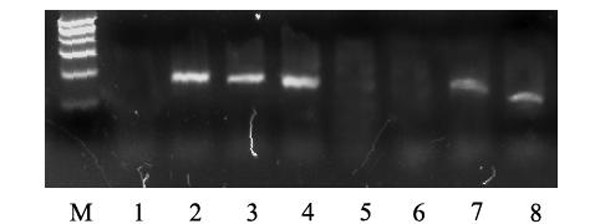
**Identification of the transcription start site for the mouse *CS1 *and *CS2 *genes by RT-PCR**. Four different forward primers and a common reverse primer were used for each gene as shown in Table 1. Lanes 1–8 indicate the results of RT-PCR using the forward primers CS1RT1L, CS1RT2L, CS1RT3L, CS1RT4L, CS2RT1L, CS2RT2L, CS2RT3L, CS2RT4L, and the corresponding reverse primers CS1RTR and CS2RTR, respectively.

To further characterize the transcriptional control of the mouse *CS-1 *and *CS-2 *genes, we first determined their respective transcription start sites. RT-PCR analysis was performed using total RNA extracts of C2C12 cells and four different forward primers and one reverse primer for each gene. As shown in Table 1, the reverse primer is located in exon 1 and the different forward primers are located around the putative transcription start sites. The results of these analyses show that the first three primer pairs of *CS-1*, and first two for *CS-2*, successfully yielded PCR products. This indicates that the sequences contained within these oligonucleotides form part of the mRNA product of each gene (Fig. 1). We concluded that the TSS sites of mouse *CS-1 *and *CS-2 *are located at -191 to -176 and -82 to -74, respectively. The nucleotide immediately upstream of the translation initiation codon (ATG) is denoted as -1.

### Transcriptional regulation of *CS-1 *and *CS-2 *during myogenic differentiation

**Figure 2:**
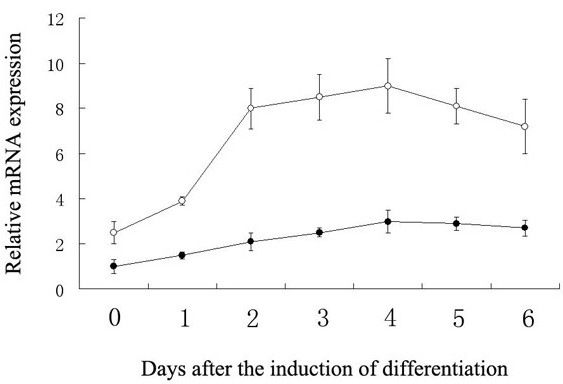
**Real-time PCR analysis of *CS1 *and *CS *2 mRNA expression during the myogenic differentiation of C2C12 cells (day 0 to day 7)**. Values are expressed as ratios of the basal transcription levels on culture day 0 for *CS2*.

Real-time PCR analysis was performed to determine the relative mRNA expression levels of both the mouse *CS-1 *and *CS-2 *genes during myoblast differentiation in C2C12 cells. Specific primers corresponding to the two genes were designed and the housekeeping gene *β-actin *was used as a control. We subsequently found that the *CS-1 *and *CS-2 *transcript levels were both low at the myoblast stage. *CS-1 *mRNA expression increases markedly, however, during the first two days of differentiation and is then maintained at relatively abundant levels throughout this process. In contrast, the *CS-2 *transcript levels remain low throughout myogenic differentiation (Fig. 2).

### Promoter analysis of the mouse *CS-1 *and *CS-2 *gene

**Figure 3:**
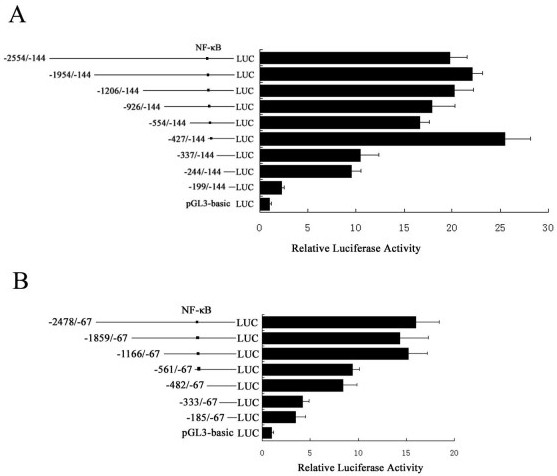
**Reporter analysis of the mouse *CS1 *and *CS2 *gene promoters**. A. Reporter analysis of mouse *CS1 *gene promoter deletion constructs. B. Reporter analysis of mouse *CS2 *gene promoter deletion constructs. The 5'-deleted promoter segments were generated as described in Materials and Methods. C2C12 cells were co-transfected with various promoter regions fused to firefly luciferase and with a Renilla luciferase expression control vector. The resulting firefly luciferase activity was then normalized to Renilla luciferase activity and the relative values are presented as the fold-increase over the activity of the promoterless pGL3-basic vector. The length of each 5'-deletion fragment is calculated relative to the translation initiation ATG codon and is indicated to the left of each bar. The presence of putative NF-κB binding elements is also indicated. Values represent the mean ± SD of three independent experiments.

To test the minimal region required for promoter activity within the upstream *CS-1 *and *CS-2 *sequences, fragments corresponding to the regions -2554 to -144 of *CS-1 *and -2478 to -67 of *CS-2 *(relative to the ATG initiation codon), were inserted into the pGL3-basic vector and luciferase assays were performed. A significant increase in luciferase activity was observed in the C2C12 cell line (almost 20-fold and 16-fold increases following transfection with the promoter-luciferase vectors pCS1-2554/-144 and pCS2-2478/-67, respectively) compared with cells transfected with the empty vector (Fig. 3). Sequence analysis of these two promoter segments revealed that the flanking region harbours potential binding sites for multiple transcription factors including Sp1 and AP-1, in addition to the muscle specific transcription factors MEF-2 and MyoD. However, no TATA-boxes are present in these regions.

We next generated a series of deletion mutants for the *CS-1 *and *CS-2 *promoters via PCR-based approaches using the pCS1-2554/-144 and pCS2-2478/-67 constructs as templates. The amplified mutant fragments were then subcloned into the pGL3-basic vector and our results are shown in Figure 3. For the *CS-1 *promoter fragments, the deletion constructs pCS1-1954/-144 and pCS1-1206/-144 display high promoter activity. Further deletions at positions -926 (pCS1-926/-144) and -554 (pCS1-554/-144) resulted in a gradual decrease in promoter activity. However, the highest promoter activity levels were observed for the pCS1-427/-144 construct, suggesting that there are inhibitory elements within the region -554 to -427. Deletions at positions -337(pCS1-337/-144) and -244(pCS1-244/-144) also reduce promoter activity dramatically, whereas the pCS1-199/-144 construct shows almost no luciferase activity (Fig. 3A). Taken together, these reporter data indicate that the core region of the basal promoter of mouse *CS-1 *gene is located within the region -427 to -337. For the *CS-2 *promoter, the deletion constructs pCS2-1859/-67, pCS2-1166/-67 and pCS2-561/-67 display high promoter activity with only modest differences between them, suggesting that each harbours the core elements necessary for the basal promoter function of the mouse *CS-2 *gene. The promoter activity of pCS2-482/-67, PCS2-333/-67 and pCS2-185/-67 decreases gradually to relatively low levels and thus further defines the core region from -561 to -185 (Fig. 3B).

For our initial characterization of these two promoters, we searched for the presence of a consensus slow upstream regulatory element (SURE) and fast intronic regulatory element (FIRE), which have been shown to drive fibre-specific gene transcription [8]. However, neither of these sites was found to be present in the two respective promoter regions after careful analysis using TESS software. We then focused on the association of other transcription factors with the two gene promoters, such as the role of calcineurin signalling factors in activating the slow-fibre specific promoter of *CS-1*. It was noticeable that the pCS1-427/-144 construct exhibited maximum activity and that deletion of the region -427 to -337 of *CS-1 *5' flanking sequence reduced the promoter activity dramatically, indicating that a strongly positive element is located in this region. A putative binding site for the NF-κB transcription factor was detectable in this region using high-stringency analysis of the TESS database. However, a putative binding site for NF-κB was also assigned to the core region of *CS-2 *promoter in these searches. Since our reporter assay findings raised the possibility that transactivation of the mouse *CS-1 *and *CS-2 *gene may be achieved through NF-κB binding elements present in their core promoters, we attempted to determine which of these two potential NF-κB binding sites was authentic during this activation event.

### Confirmation of NF-κB binding to the *CS-1 *promoter by EMSA

**Figure 4:**
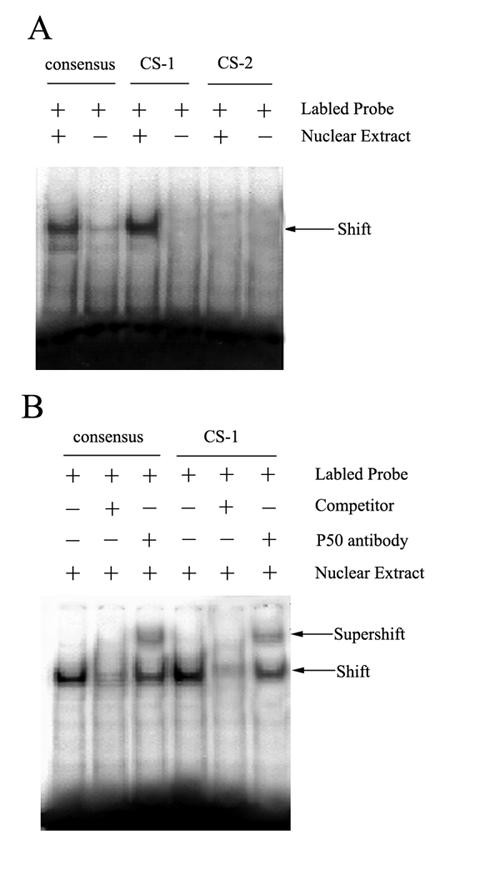
**EMSA analysis of the NF-κB binding sites in the mouse *CS*-1 and *CS-2 *promoters**. A. ^32^P labelled oligonucleotide probes for the NF-κB consensus, NF-κB *CS-1 *and NF-κB *CS-2 *binding sites were incubated with C2C12 myotube nuclear extracts. B. Competition experiments were performed using a 100-fold excess of unlabeled NF-κB consensus and NF-κB *CS-1 *probes. Supershift assays were performed with anti-P50 antibody. Arrows indicate the resulting bandshifts.

We synthesized specific oligonucleotides containing the NF-κB elements present in the *CS-1 *and *CS-2 *promoters and tested them with an NF-κB consensus sequence control in EMSA experiments with nuclear extracts from C2C12 myotubes. As shown in Figure 4A, incubation of C2C12 nuclear extracts with both the NF-κB consensus sequence and NF-κB-CS1 sequence produced a DNA-protein band shift. In contrast, the NF-κB-CS2 oligonucleotide probe failed to form such a complex in this experiment. These DNA-protein complexes were determined to be specific to the NF-κB sites by successful competition assays using excess unlabeled consensus and NF-κB-CS1 oligonucleotides (Fig. 4B). To confirm the binding of NF-κB family members to the NF-κB-CS1 sequence, these EMSA reactions were further incubated with antibodies raised against P50 of NF-κB. As shown in Figure 4B, the addition of this antibody resulted in a supershifted complex in addition to the DNA-protein band. These data confirm the presence of the NF-κB family member, P50, in the nuclear protein complex that binds the NF-κB binding site of the *CS-1 *promoter.

### The inhibition of NF-κB downregulates *CS-1 *promoter activity but does not affect *CS-2*

**Figure 5:**
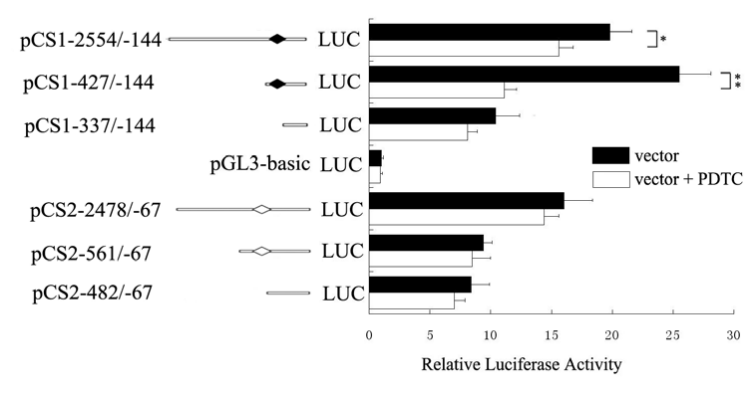
**The differential effects of PDTC on the activity of mouse *CS1 *and *CS2 *gene promoter fragments harbouring 5' deletions**. At 24 hours after transfection with reporter constructs, C2C12 cells were treated with PDTC (10 μM) for a further 24 hours, and the firefly luciferase activities were measured and normalized to the Renilla luciferase controls. The data values are the fold-increases (mean ± SD) in luciferase activity for each treatment over the basic luciferase vector sample activity without PDTC, and are the results of triplicate experiments. The statistical significance of the differences between the basal transcription and PDTC exposure values were analyzed by the Student's t-test, * P < 0.05, ** P < 0.001.

To further illustrate the biological importance of NF-κB in the regulation of *CS-1*, we inhibited NF-κB transactivation by treatment with pyrrolidine dithiocarbamate (PDTC), a proven free radical scavenger that accelerates IκB dissociation with a resulting block in NF-κB transport to the nucleus and subsequent binding to DNA. As shown in Figure 5, when C2C12 cells were transfected with the 2.5-kb wild type *CS-1 *and *CS-2 *promoter luciferase reporter plasmids, a 20-fold and 16-fold increase in reporter activity was observed, respectively. The treatment of these cells with PDTC induces a modest decrease in luciferase activity for both wild-type reporter plasmids, and had no impact on the empty vector control (Fig. 5). Transfection of the shorter *CS-1 *and *CS-2 *promoter fragment reporters (pCS1-427/-144 and pCS2-561/-67), both containing their respective putative NF-κB elements, results in a 25-fold and 10-fold increase in luciferase activity, respectively. In this same experiment, PDTC treatment induced a 2–3-fold decrease in luciferase activity for *CS-1 *but no apparent changes were evident for the *CS-2 *reporter activities. However, when cells were transfected with the *CS-1 *and *CS-2 *reporter plasmids containing proximally shorter promoter fragments (pCS1-337/-144 and pCS2-482/-67), we again detected a large increase in luciferase activity compared with the empty vector, but PDTC has no effects. These data suggest that the NF-κB binding element located in the region -427 to -337 of CS-1 promoter plays an important role in the transcriptional activity of this gene.

### The effects of NF-κB, NFAT and MEF2 overexpression upon the *CS-1 *and *CS-2 *promoters

Since putative NFAT and MEF2 binding elements are located within the *CS-1 *and *CS-2 *promoters, the sensitivities of the NFAT and MEF2 sites, as well as NF-κB site, to the overexpression of specific transcription factors were also determined. First, the 2.5-kb wild type *CS-1 *and *CS-2 *promoters were cotransfected with pReceiver-NF-κB1, which constitutively expresses the NF-κB subunit gene p50. The overexpression of p50 significantly induces CS-1 promoter activity and also the positive NF-κB reporter control (pNF-κB-Luc). A further 5' deletion of the NF-κB site totally abolishes the response to NF-κB overexpression, reconfirming the authenticity of this site. However, the activity of CS-2 promoter was found to be increased by 1.7-fold when cotransfected with NF-κB in these experiments, indicating that CS-2 is not independent of NF-κB. This partially contradicts the results of our gel shift and drug treatment experiments. Hence, other NF-κB elements may exist in the CS-2 promoter or it may be triggered by other factors induced by NF-κB.

**Figure 6:**
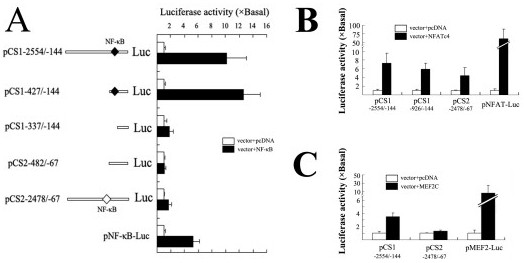
**The activation of CS-1 and CS-2 promoters by different signalling molecules**. A. Deleted promoter constructs of CS-1 and CS-2 were used in the NF-κB p50 cotransfection analysis. A schematic representation of these two promoters is shown. These vectors were cotransfected with pReceiver-NF-κB1. B. A range of different truncated promoter constructs (pCS1-2554/-144, pCS1-926/-144 and pCS2-2478/-67) were cotransfected with pcDNA-NFATc4. A consensus NFAT element in the region -1206 to -926 was removed in the pCS1-926/-144 vector. C. The two wild type calsarcin promoters were cotransfected with pReceiver-MEF2C. The pNF-κB-Luc, pNFAT-Luc and pMEF2-Luc vectors were used as controls. Values are expressed as ratios of the basal activity (resulting from cotransfection with an empty pcDNA3.1 vector) and represent the mean ± SD for two sets of independent experiments, each performed in triplicate. (Note: comparison of luciferase activity between different vectors is not valid.)

As a preliminary test for the role of the calcineurin-NFAT-MEF2 signalling pathway [8] in the regulation of the calsarcin promoters, we next determined whether the *CS-1 *or *CS-2 *promoters are sensitive to the overexpression of NFAT or MEF2 in C2C12 cells. When cotransfected with the NFATc4 expression plasmid (pcDNA-NFATc4), both of the promoters show enhanced transcription activities, but to different extents (CS-1, 7.2 ± 2.3 fold; CS-2, 4.4 ± 1.8 fold; and pNFAT-Luc, 68.8 ± 13.6 fold). However, only a moderate decrease in promoter activity was observed after 5' deletion of the -1206 to -926 region of *CS-1 *promoter which harbours a NFAT consensus element, indicating that other putative binding motifs are present. Similarly, MEF2C overexpression was achieved by cotransfection of pReceiver-MEF2C with the luciferase reporter vector. This overexpression induces the activity of the *CS-1 *promoter by 3.5-fold but has only marginal effects upon the *CS-2 *promoter. This indicates that MEF2 also contributes to the differential transcription of the calsarcins in different fibres. However, it is noteworthy that both promoters contain several putative MEF2 binding elements (Fig. 6).

## Discussion

The differences in the contractile and biochemical properties of slow- and fast-twitch myofibres are determined by the selective transcription of genes coding for contractile proteins and metabolic enzymes in these muscles. These distinctive programs of gene expression are further controlled by various regulatory elements and pathways, such as the calcineurin signalling pathway, which still require further elucidation. One approach to further understanding the molecular mechanisms controlling muscle diversification and plasticity is to identify the DNA regulatory sequences that control the fibre-type-specific expression of contractile protein genes. The study of the transcriptional regulation of fibre-type-specific calsarcins, which is also a family of calcineurin-interaction proteins, would contribute to our understanding of the role of calcineurin in muscle fibre differentiation.

In our present study, we determined by RT-PCR that the transcription start sites of the mouse *CS-1 *and *CS-2 *genes are in the regions -191 to -176 and -82 to -74 upstream of the ATG initiation codon, respectively. We then isolated the promoter regions of these mouse calsarcin genes by PCR. In order to identify their cis-regulatory elements, a series of deletion constructs from the 5'-flanking regions were analyzed. For *CS-1*, we found that a construct containing a putative NF-κB site demonstrated the highest promoter activity levels. We thus speculated that this potential binding site for NF-κB and other putative transcription factor sites in this region would be important for the expression of the *CS-1 *gene. A significant decrease in *CS-1 *promoter reporter activity was indeed observed after the further deletion of this NF-κB element. It is thus quite likely that the NF-κB site in the *CS-1 *promoter contributes greatly to its transactivation. However, a putative NF-κB site is also located in the core region of *CS-2 *promoter, but our deletion reporter analysis did not reveal any significant involvement of this element in the transcriptional control of *CS-2*.

Gel retardation assays were performed to validate the binding of NF-κB to the core promoter regions of *CS-1 *and *CS-2*. A *CS-1 *NF-κB sequence probe was found to induce a bandshift when incubated with nuclear extracts from C2C12 myotubes in a similar manner to the NF-κB consensus sequence control. However, no such band was evident when these extracts were incubated with a *CS-2 *NF-κB sequence. Competition and supershift EMSA experiments further confirmed that the NF-κB element in the core region of the *CS-1 *promoter is recognized by the P50 subunit of NF-κB. Moreover, treatment with an NF-κB inhibitor (PDTC) resulted in a dramatic reduction in reporter gene activity of the deletion fragment pCS1-427/-144, which harbours the NF-κB binding element, with a modest decrease evident in the activity of the wild-type *CS-1 *promoter in this experiment. In contrast, none of the additional reporter constructs showed any PDTC responsiveness. In addition, the region located upstream of the NF-κB element in the *CS-1 *promoter appears to contain suppressive elements that reduce the effects of this inhibitor. Overexpression of p50 of NF-κB increases the activity of the *CS-1 *promoter significantly, but no real change was evident for *CS-2 *in this analysis. Taken together, these data suggest that NF-κB plays a critical role in the transcriptional activity of the mouse *CS-1 *gene, but does not play the same role for *CS-2*.

Several studies have previously demonstrated that calcineurin stimulates slow muscle gene promoters and slow fibre differentiation, both in culture and *in vivo*, via the calcineurin-NFAT signalling pathway [11-13]. This mechanism has also been found to be active for many type-specific genes harbouring a SURE element (containing the NFAT sequence) in the slow promoter, and a FIRE element in the fast promoter [8,14,15]. However, no effects of calcineurin on slow muscle gene expression have been observed in other studies based either on CsA treatment [16] or on the overexpression of calcineurin and NFAT [17]. In our present study, sequencing analyses did not reveal any consensus SURE or FIRE elements within the *CS-1 *and *CS-2 *gene promoters, but putative NFAT binding sites were evident in both of these promoters by TESS analysis. To determine whether NFAT or MEF2 are involved in the transcription regulation of these genes, we overexpressed NFATc4 and MEF2C and compared the resulting the promoter activities with their corresponding basal levels. The *CS-1 *promoter was found to be upregulated by either NFATc4 or MEF2C, whereas *CS-2 *appears to be more resistant to this activation, indicating that these factors may also play a role in the transcriptional control of these genes. These findings also raised the possibility that calcineurin-NFAT signalling pathway participates, at least partly, in the regulation of the calsarcin promoters. It is thought that no single regulatory pathway can dominate the regulation of each muscle-specific gene [18], and it is considered likely that different pathways act cooperatively in the exclusive expression of a specific gene in slow or fast fibres. Interestingly, an important signalling and functional link between the calcineurin-NFAT and calcineurin-NF-κB pathways has been established by stable expression of constitutively active calcineurin in muscle C2C12 cells. This proposed model suggests dual actions for calcineurin upon terminal differentiation with opposing phenotypes, through its transient effects on NFAT and prolonged effects on NF-κB, respectively [19]. Studies of CS-1 knockout mice also demonstrate that CS-1 negatively modulates the functions of calcineurin and it will therefore be important to determine whether the transcription of the calsarcins is also subject to regulation by calcineurin signalling.

NF-κB has been implicated as a modulator of muscle catabolism and as a putative regulator of protein breakdown from existing fibres [20]. However, the complexities of the NF-κB signalling mechanisms in muscle are just now beginning to be elucidated. Significantly also, disuse muscle atrophy is associated with the activation of the NF-κB pathway [21,22], and disruption of the NF-κB1 gene inhibits skeletal muscle atrophy mainly in fast fibres [23]. The slow fibres in this case are atrophied to the same extent as their normal counterparts, indicating that there are different roles for NF-κB1 in slow and fast fibres during muscle atrophy [23]. In our current study, we have shown that the slow fibre specific *CS-1*, but not fast fibre specific *CS-2*, is a putative downstream target gene of NF-κB. Other factors related to calcineurin, such as NFAT and MEF2, were also found to participated in the regulation of these two gene promoters. In other words, the activation of this fibre-specific transcription appears to be mediated by a combinatorial mechanism involving different pathways and multiple factors. However, this mechanism was only tested in C2C12 myotubes in our present study, and whether further investigations will be needed *in vivo*. In addition, as it has also been speculated that the overexpression of a specific signalling factor may impact upon multiple muscle genes rather than just one targeted gene, we cannot yet exclude the possibility that the induced promoter activity is non-specific or is due to system effects of the enhanced pathways which were analysed. Hence, whether this selective transactivation of calsarcins by NF-κB as well as other factors contributes to the atrophy discrepancies or the opposing phenotypes seen in slow and fast fibres needs further study.

## Conclusion

Our current results provide the first insights into the mechanisms regulating the transcription of members of the mouse calsarcin gene family, which are differentially expressed in slow- or fast-twitch muscle fibre cells. We have defined the proximal promoter region of both the *CS-1 *and *CS-2 *genes and further identified an NF-κB binding site in *CS-1 *core region, which contributes significantly to the transcriptional control of this gene. Additionally, our data reveal that NF-κB has effects upon *CS-1*, but not *CS-2*, promoter function. In addition, the results of our overexpression analysis suggest that NFAT and MEF2 are also involved in the transcriptional regulation of these two genes. Our data thus suggest that NF-κB plays an important role in the regulation of calsarcin expression and that different signalling pathways may cooperate with each other in a complex network of calsarcin transcriptional regulation. These findings provide some important clues that will enable further analysis of the role of calsarcin gene family in fibre-type specificity via calcineurin signalling.

## Methods

### Cell culture

C2C12 mouse skeletal myoblasts were cultured in DMEM/high glucose supplemented with 20% FBS, 100 units/ml penicillin and 100 μg/ml streptomycin and maintained at 37°C in 5% CO_2_. The differentiation of C2C12 myoblasts into myotubes was achieved by the addition of differentiation medium (DMEM supplemented with 2% horse serum) for up to 6 days with medium changes every 2 days.

### RT-PCR and real-time PCR

C2C12 cells were cultured in six-well plates and harvested for RNA extraction according to the time course of differentiation. Total RNA was isolated using Trizol reagent (Invitrogen) according to the manufacturer's instructions. cDNA was then reverse-transcribed from normalized RNA using random hexamer primers and M-MLV reverse transcriptase (Promega). To identify the initial transcription start site of the *CS-1 *and *CS-2 *genes, primer sets were designed for use with an RT-PCR kit (TaKaRa), according to the manufacturer's instructions [see Additional file 1]. Real-time PCR was performed using the SYBR Premix Ex Taq Kit (TaKaRa) with cycling conditions consisting of an initial 5 min at 95°C, followed by 35 cycles comprising 15 sec at 95°C, 20 sec at 61°C, 20 sec at 72°C, and fluorescence acquisition at 83°C for 1 sec. Purified T-easy vectors (Promega) containing the target promoter fragments were serially diluted (undiluted, 1/4, 1/16, 1/64 and 1/256) to construct standard curves for determining the optimal amplification conditions. PCR was then performed in triplicate and the gene expression levels were quantified relative to the expression of *β-actin *using Gene Expression Macro software (Bio-Rad), employing an optimized comparative Ct (ΔΔCt) value method. Dissociation curves were generated to ensure a single amplicon had been produced.

### Plasmid constructs

The mouse *CS-1 *and *CS-2 *gene sequences were obtained from GenBank (NC_000069 and NC_000080) and were employed to designed primers that would isolate their 5'-flanking regions. For this purpose, we utilized LA Taq (TaKaRa), and cloned the amplicons into the T-Easy vector for subsequent manipulation. A series of constructs containing variable lengths of the mouse *CS-1 *and *CS-2 *promoter was then generated by PCR using forward primers for sequences located at varying distances from the major transcription start site and including an MluI recognition sequence to facilitate ligation. The primers pCS1-144R and pCS2-67R, both containing a XhoI recognition sequence, were used as the common reverse primer for their respective genes and had 5' ends which were located within the first exon in each case [see Additional file 1]. Amplified fragments were then inserted into the multiple cloning site of the pGL3-basic vector to generate luciferase reporter constructs. The NFkB1 and MEF2C overexpression vectors were pReceiver-NF-κB1 and pReceiver-MEF2C, encoding p50 and MEF2C (GeneCopoeia). The coding region of NFATc4 gene was cloned into the *Xba*I-*Bam*HI site of pcDNA3.1 to construct the NFAT expression vector pcDNA-NFATc4. The pNF-κB-Luc and pNFAT-Luc reporter plasmids were purchased from Stratagene. The pMEF2-Luc reporter construct contains three tandem copies of the consensus binding element (GGCTCTAAAAATAGCCCCC) upstream of the basic promoter element (TATA box) and the luciferase gene.

### Transfections and drug treatments

The cells were seeded into 96-well plates at an initial density of 60–80% and cultured overnight to ensure adhesion and spreading. Co-transfections were then performed using 0.5 μl of Lipofectamine 2000 reagent (Invitrogen) with 200 ng of the appropriate firefly luciferase plasmid DNA, and 20 ng of pRL-TK plasmid DNA (Promega) as an internal control. For cotransfection analyses, the levels of reporter plasmids were kept constant, but 100 ng of the relevant expression vector or empty pcDNA3.1 were added. The pGL3-Control vector (Promega) was used as a positive control. The transfection medium was removed and replaced with growth medium after 4 to 6 hours. The differentiation medium was then added 12 hours after transfection. Firefly and Renilla luciferase activities were measured at 48 hours post-transfection using the Dual-Glo Luciferase assay system (Promega) and a TD20/20 luminometer (Turner Designs). The transfection efficiencies in each case were normalized using the Renilla luciferase activity levels and each construct was tested in triplicate in a minimum of three independent experiments. At 24 hours after transfection, the cells were treated with 10 μM PDTC (pyrrolidine dithiocarbamate, Sigma, St. Louis, MO) and cultured for another 24 hours before assaying for luciferase activity.

### Gel Mobility Shift Assay

Nuclear extracts were prepared from cultured C2C12 myotubes using the NE-PER Nuclear and Cytoplasmic Extraction kit (Pierce). The protein concentrations were determined using the Bradford assay (Bio Rad). Nuclear protein extracts (3–6 μg) were then incubated in 10 μl of binding buffer (10 mM Tris-HCl, pH 7.5, 4% glycerol, 1 mM MgCl_2_, 0.5 mM EDTA, 0.5 mM DTT, 50 mM NaCl, 50 μg/ml of double-stranded poly [dI-dC]) at room temperature for 10 min before adding 35 fmol of the appropriate end-labelled double-stranded oligonucleotide [see Additional file 1]. DNA labelling was performed using T4 polynucleotide kinase and [γ-32P] ATP. Formation of the DNA-protein complexes was allowed to proceed for 20 min at room temperature before stopping the reaction by the addition of 1 μl gel loading dye. For competition experiments, a 100-fold excess of unlabeled double-stranded oligonucleotide was added to the reaction. For supershift experiments, 2 μg of antibody against P50 of NF-kappaB (Santa Cruz Biotechnology, Santa Cruz) was added to the reaction mixture and incubated with the nuclear extracts on ice for 15 min before the addition of the probe, followed by a further incubation on ice for 20 min. The samples were then electrophoresed on 4% nondenaturing polyacrylamide gels in 0.5 × TBE buffer (45 mM Tris, 45 mM borate, 1 mM EDTA, pH 8.3) and the gels were dried and exposed to X-ray film at -70°C with intensifying screens.

### DNA sequence analysis

DNA sequences were analyzed for the presence of putative transcription factor binding sites using the TESS (Transcription Element Search System) web tool available from the Computational Biology and Informatics Laboratory at the University of Pennsylvania.

## Authors' contributions

HW performed the experiments and drafted the manuscript. SY participated in the design of the study and assisted in statistical analysis. EY helped in vectors construction experiments. ZZ, YM and SF supplied technical expertise and assisted in editing the manuscript. KL designed, coordinated and helped to draft the manuscript. All the authors read and approved the final manuscript.

## Supplementary Material

Additional File 1 Oligonucleotides. Oligonucleotides used as primers for vector construction and RT-PCR, and as probes for EMSA. Click here for file

## References

[B1] Wang J, Shaner N, Mittal B, Zhou Q, Chen J, Sanger JM, Sanger JW (2005). Dynamics of Z-band based proteins in developing skeletal muscle cells. Cell Motil Cytoskeleton.

[B2] Frey N, Richardson JA, Olson EN (2000). Calsarcins, a novel family of sarcomeric calcineurin-binding proteins. Proc Natl Acad Sci U S A.

[B3] Faulkner G, Pallavicini A, Comelli A, Salamon M, Bortoletto G, Ievolella C, Trevisan S, Kojic S, Dalla Vecchia F, Laveder P, Valle G, Lanfranchi G (2000). FATZ, a filamin-, actinin-, and telethonin-binding protein of the Z-disc of skeletal muscle. J Biol Chem.

[B4] Takada F, Vander Woude DL, Tong HQ, Thompson TG, Watkins SC, Kunkel LM, Beggs AH (2001). Myozenin: an alpha-actinin- and gamma-filamin-binding protein of skeletal muscle Z lines. Proc Natl Acad Sci U S A.

[B5] Ahmad F, Gonzalez O, Ramagli L, Xu J, Siciliano MJ, Bachinski LL, Roberts R (2000). Identification and characterization of a novel gene (C4orf5) located on human chromosome 4q with specific expression in cardiac and skeletal muscle. Genomics.

[B6] Frey N, Olson EN (2002). Calsarcin-3, a novel skeletal muscle-specific member of the calsarcin family, interacts with multiple Z-disc proteins. J Biol Chem.

[B7] Wang H, Zhu Z, Wang H, Yang S, Mo D, Li K (2006). Characterization of different expression patterns of calsarcin-1 and calsarcin-2 in porcine muscle. Gene.

[B8] Chin ER, Olson EN, Richardson JA, Yang Q, Humphries C, Shelton JM, Wu H, Zhu W, Bassel-Duby R, Williams RS (1998). A calcineurin-dependent transcriptional pathway controls skeletal muscle fiber type. Genes Dev.

[B9] Schulz RA, Yutzey KE (2004). Calcineurin signaling and NFAT activation in cardiovascular and skeletal muscle development. Dev Biol.

[B10] Frey N, Barrientos T, Shelton JM, Frank D, Rutten H, Gehring D, Kuhn C, Lutz M, Rothermel B, Bassel-Duby R, Richardson JA, Katus HA, Hill JA, Olson EN (2004). Mice lacking calsarcin-1 are sensitized to calcineurin signaling and show accelerated cardiomyopathy in response to pathological biomechanical stress. Nat Med.

[B11] Allen DL, Sartorius CA, Sycuro LK, Leinwand LA (2001). Different pathways regulate expression of the skeletal myosin heavy chain genes. J Biol Chem.

[B12] Chakkalakal JV, Stocksley MA, Harrison MA, Angus LM, Deschenes-Furry J, St-Pierre S, Megeney LA, Chin ER, Michel RN, Jasmin BJ (2003). Expression of utrophin A mRNA correlates with the oxidative capacity of skeletal muscle fiber types and is regulated by calcineurin/NFAT signaling. Proc Natl Acad Sci U S A.

[B13] D'Andrea M, Pisaniello A, Serra C, Senni MI, Castaldi L, Molinaro M, Bouche M (2006). Protein kinase C theta co-operates with calcineurin in the activation of slow muscle genes in cultured myogenic cells. J Cell Physiol.

[B14] Calvo S, Vullhorst D, Venepally P, Cheng J, Karavanova I, Buonanno A (2001). Molecular dissection of DNA sequences and factors involved in slow muscle-specific transcription. Mol Cell Biol.

[B15] Lee HH, Choi RC, Ting AK, Siow NL, Jiang JX, Massoulie J, Tsim KW (2004). Transcriptional regulation of acetylcholinesterase-associated collagen ColQ: differential expression in fast and slow twitch muscle fibers is driven by distinct promoters. J Biol Chem.

[B16] Biring MS, Fournier M, Ross DJ, Lewis MI (1998). Cellular adaptations of skeletal muscles to cyclosporine. J Appl Physiol.

[B17] Swoap SJ, Hunter RB, Stevenson EJ, Felton HM, Kansagra NV, Lang JM, Esser KA, Kandarian SC (2000). The calcineurin-NFAT pathway and muscle fiber-type gene expression. Am J Physiol Cell Physiol.

[B18] Spangenburg EE, Booth FW (2003). Molecular regulation of individual skeletal muscle fibre types. Acta Physiol Scand.

[B19] Alzuherri H, Chang KC (2003). Calcineurin activates NF-kappaB in skeletal muscle C2C12 cells. Cell Signal.

[B20] Li YP, Reid MB (2000). NF-kappaB mediates the protein loss induced by TNF-alpha in differentiated skeletal muscle myotubes. Am J Physiol Regul Integr Comp Physiol.

[B21] Hunter RB, Stevenson E, Koncarevic A, Mitchell-Felton H, Essig DA, Kandarian SC (2002). Activation of an alternative NF-kappaB pathway in skeletal muscle during disuse atrophy. Faseb J.

[B22] Kumar A, Boriek AM (2003). Mechanical stress activates the nuclear factor-kappaB pathway in skeletal muscle fibers: a possible role in Duchenne muscular dystrophy. Faseb J.

[B23] Hunter RB, Kandarian SC (2004). Disruption of either the Nfkb1 or the Bcl3 gene inhibits skeletal muscle atrophy. J Clin Invest.

